# Enhancing vector control: AI-based identification and counting of *Aedes albopictus* (Diptera: Culicidae) mosquito eggs

**DOI:** 10.1186/s13071-024-06587-w

**Published:** 2024-12-18

**Authors:** Minghao Wang, Yibin Zhou, Shenjun Yao, Jianping Wu, Minhui Zhu, Linjuan Dong, Dunjia Wang

**Affiliations:** 1 Key Laboratory of Geographic Information Science, Ministry of Education, Shanghai, China; 2 School of Geographic Sciences, East China Normal University, Shanghai, China; 3 Minhang Center for Disease Prevention and Control, Shanghai, China; 4 Key Laboratory of Spatial-Temporal Big Data Analysis and Application of Natural Resources in Megacities, Ministry of Natural Resources, Shanghai, China; 5 Institute of Cartography, East China Normal University, Shanghai, China

**Keywords:** *Aedes albopictus*, Mosquito eggs, Faster R-CNN, Segment Anything Model, Ovitraps, Egg identification and counting

## Abstract

**Background:**

Dengue fever poses a significant global public health concern, necessitating the monitoring of *Aedes* mosquito population density. These mosquitoes serve as the disease vectors, making their surveillance crucial for dengue prevention. The objective of this study was to address the difficulty associated with identifying and counting mosquito eggs of wild strains during the monitoring of *Aedes albopictus* (Diptera: Culicidae) density via ovitraps in field surveys.

**Methods:**

We constructed a dataset comprising 1729 images of *Ae. albopictus* mosquito eggs from wild strains and employed the Segment Anything Model to enhance the applicability of the detection model in complex environments. A two-stage Faster Region-based Convolutional Neural Network model was used to establish a detection model for *Ae. albopictus* mosquito eggs. The identification and counting process involved applying the tile overlapping method, while morphological filtering was employed to remove impurities. The model’s performance was evaluated in terms of precision, recall, and F1 score, and counting accuracy was assessed using *R*-squared and root mean square error (RMSE).

**Results:**

The experimental results revealed the model’s remarkable identification capabilities, achieving precision of 0.977, recall of 0.978, and an F1 score of 0.977. The *R*-squared value between the actual and identified egg counts was 0.997, with an RMSE of 1.742. The average detection time for a single tile was 0.48 s, which was more than 10 times as fast as the human–computer interaction method in counting an entire image.

**Conclusions:**

The model demonstrated excellent performance in recognizing and counting *Ae. albopictus* mosquito eggs, indicating great application potential. This study offers novel technological support for enhancing vector control effectiveness and public health standards.

**Graphical Abstract:**

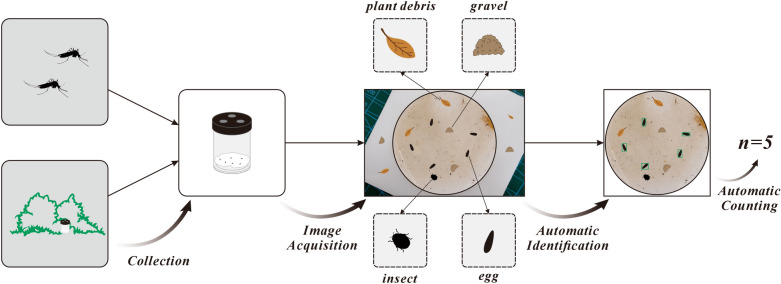

## Background

Dengue fever, a febrile illness caused by the dengue virus transmitted by *Aedes aegypti* or *Aedes albopictus* (Diptera: Culicidae) mosquitoes [1], is a prevalent and rapidly spreading mosquito-borne disease [2]. Characterized by high incidence and strong infectiousness [3], dengue fever poses a severe threat to public health and safety, with 2.5 billion people at risk of dengue infection annually [4], presenting significant challenges to both economic development [5] and public health [6]. With the advancement of global phenomena [7] such as air travel, maritime trade [8], climate change [9, 10], and urbanization [11, 12], mosquitoes can travel long distances in a short time, leading to an increasing risk of dengue infection and expanding geographic distribution annually [13, 14]. Although a dengue fever vaccine has been widely authorized, its dissemination has been slow due to safety concerns identified in recipients [15], along with the absence of licensed antiviral drugs currently for treating dengue infection [16]. Therefore, preventing dengue infection is an urgent public health issue that needs to be addressed globally.

Vector control has proven to be an effective method for preventing dengue infection [17]. By limiting mosquito–human interactions and controlling mosquito populations, the spread of the virus can be curtailed. The effectiveness of vector control depends on the monitoring of vectors. Research has shown that the reproductive capacity of female mosquitoes can provide valuable information for mosquito vector monitoring, such as estimating population density [18] and identifying potential breeding sites [19]. Monitoring *Aedes* mosquitoes can effectively guide the allocation of resources to control mosquito density, thereby preventing the spread of dengue fever [20]. Among the various non-chemical methods for monitoring *Aedes* mosquitoes, ovitraps are considered an effective way to monitor mosquito populations because of their low cost, ease of operation, and standardization [21], and have been widely applied in regions such as Malaysia [22], Indonesia [23], and Rome [24]. Ovitraps, which collect mosquito eggs, can quantify the reproductive ability of female mosquitoes. By analyzing the number and behavior of mosquito eggs, the population size of mosquitoes can be accurately estimated, thereby assisting in decision-making for vector control [25]. However, mosquito eggs collected by ovitraps typically require identification in the laboratory using tools such as microscopes [26]. Compared with adult mosquito identification, egg identification demands greater professional skills [27, 28]. In most cases, the identification and counting of mosquito eggs are performed manually, resulting in a high workload, low efficiency, and a degree of subjective error [29, 30]. Therefore, optimizing the process of egg identification and counting, avoiding manual errors, and improving efficiency are crucial for the effective surveillance of *Aedes* mosquito density and vector control decision-making.

Previous studies have explored methods for enhancing the counting of mosquito eggs by collecting images and utilizing computer-assisted artificial identification and counting [29]. This approach, which involves human–computer interaction, is at least twice as fast as traditional manual counting via magnifiers or microscopes. While this method enables workers to perform counting remotely, it is still heavily dependent on manual labor. The widespread application of digital image processing technology further enhances the counting process [31–33]. Since RGB (red, green, blue) images provide limited computational information [34], they were usually converted to other color systems such as HSV (hue, saturation, value) [31], HSL (hue, saturation, lightness) [33], YIQ (luminance, in-phase chrominance, quadrature chrominance) [33], and International Commission on Illumination (CIE) 1976 L*a*b* (CIELAB) color space [32]. The image segmentation was then used to distinguish between mosquito eggs and the background. The determination of the threshold for image segmentation is crucial to the entire process. Manual adjustment of the segmentation threshold requires operators to have prior experience, and different images may require significantly different thresholds [35]. Additionally, the collected filter paper may darken due to bacterial or fungal growth, or soaking in sewage, complicating the determination of the threshold. To address the problem, researchers have employed optimization algorithms to determine the thresholds, such as grid search parameter optimization [31], minimizing the measures of fuzziness [33], and Gabor wavelets [36].

Rapid advancements in machine learning have also found widespread application in mosquito egg counting in recent decades [37]. For instance, an early study by Gusmão et al. [32] employed *k*-means clustering to differentiate mosquito eggs, backgrounds, and impurities based on image information from the CIELAB color system. In these studies, after the mosquito egg area is extracted through threshold segmentation, the number of mosquito eggs is determined by comparing the total pixel count occupied by the eggs to the pixel count of individual eggs. Some other studies have implemented the process using existing image processing software such as ImageJ [38], ICount [35], or self-developed tools like Egg-Counter [39] and MECVision [40], to achieve initial automated counting of mosquito eggs. All these studies base their identification and counting of mosquito eggs on the pixel scale, which has a natural advantage when egg density is high and overlapping occurs, as it does not require consideration of individual egg morphology. However, this method has certain drawbacks. When samples are collected on filter papers placed in the field, contaminants such as plant fragments, sand, and insect corpses may also be collected alongside mosquito eggs. As a result, the segmented mosquito egg pixels may contain more than just mosquito eggs, making it difficult to obtain accurate information on true positives and true negatives. Although most studies have optimized results by setting size thresholds or manually making corrections to remove impurities, this introduces uncertainty of human operations and the thresholds significantly affect its accuracy.

Another type of machine learning approach is based on object-scale operations. Image segmentation resolves the category attribution of each pixel, while object detection combines segmentation with knowledge to determine the category and location of the target [41]. AlexNet [42] has achieved a significant breakthrough in the field of image recognition, highlighting the capabilities of convolutional neural networks (CNNs), which have been widely applied in the fields of medicine and public health [43, 44]. Object detection has been used extensively for detecting and identifying various insects, such as economically significant silkworms [45] and crop-destroying pests [46]. In mosquito vector monitoring, object detection is applied to all life stages of mosquitoes (i.e., eggs, larvae, pupae, and adults), with most research focusing on larvae and adult stages. Object detection has shown significant potential in mosquito vector monitoring, whether through single-stage algorithms such as Single Shot MultiBox Detector (SSD) [47] and You Only Look Once (YOLO) [48] for detecting mosquito larvae, or two-stage algorithms like Faster Region-based Convolutional Neural Network (Faster R-CNN) for identifying adult mosquitoes [49]. Some researchers have employed object detection to identify the mosquito eggs. For example, Javed et al. [50] collected 100 macro- and microimages of laboratory strains of mosquito eggs using cameras and microscopes, respectively, and detected eggs via Mask R-CNN, with precision, recall, and F1 score above 0.9 at both the macro and micro levels. While the majority of studies are based on laboratory strains, some researchers have made efforts on wild strains. De Santana et al. [51] employed Region-based Fully Convolutional Networks (R-FCN) to identify the field-collected mosquito eggs, and the classification accuracy reached 91%. Garcia et al. [52] performed image preprocessing prior to object detection, where RGB and CIELAB information was used to distinguish between mosquito egg pixels and non-egg pixels, and small-sized objects were excluded from the process. The R-CNN was subsequently employed for detection based on the classification results, achieving a detection rate of 91% at an intersection over union (IoU) of 0.3. Although the study highlighted the removal of the background outside the filter paper prior to detection, the method primarily deals with the black areas which originated during the image acquisition with the magnification lens. Its effectiveness in removing more complex backgrounds remains to be further examined.

Despite the high precision and recall reported in these studies, several shortcomings remain. The image preprocessing process depends on manual intervention using third-party image processing software, and the parameters applied lack generalizability. Furthermore, irrespective of pixel or object scale, the majority of the collected images represent laboratory-collected mosquito eggs, which may lead to lower detection rates when identifying images collected from wild environments in real-world applications.

This study aims to address the automatic identification and counting of *Ae. albopictus* mosquito eggs from wild strains. We collected a large number of samples from field surveys for training the object detection model and standardized the image preprocessing procedures. The Segment Anything Model (SAM) [53], an artificial intelligence (AI)-based image segmentation model, was employed to effectively solve the problem of complex background interference, facilitating broader application scenarios. Then the Faster R-CNN, an AI-based object detection model, was employed to learn sufficient information about objection. For identification, we employed a tile-overlapping image-slicing method, which not only increased the pixel ratio of egg targets but also avoided the impact of mosquito eggs being segmented during the identification process. Additionally, we optimized the identification results through morphological filtering to ensure the accuracy and reliability of identification and counting. Our method demonstrated strong capabilities in recognition and counting.

## Methods

**Fig. 1:**
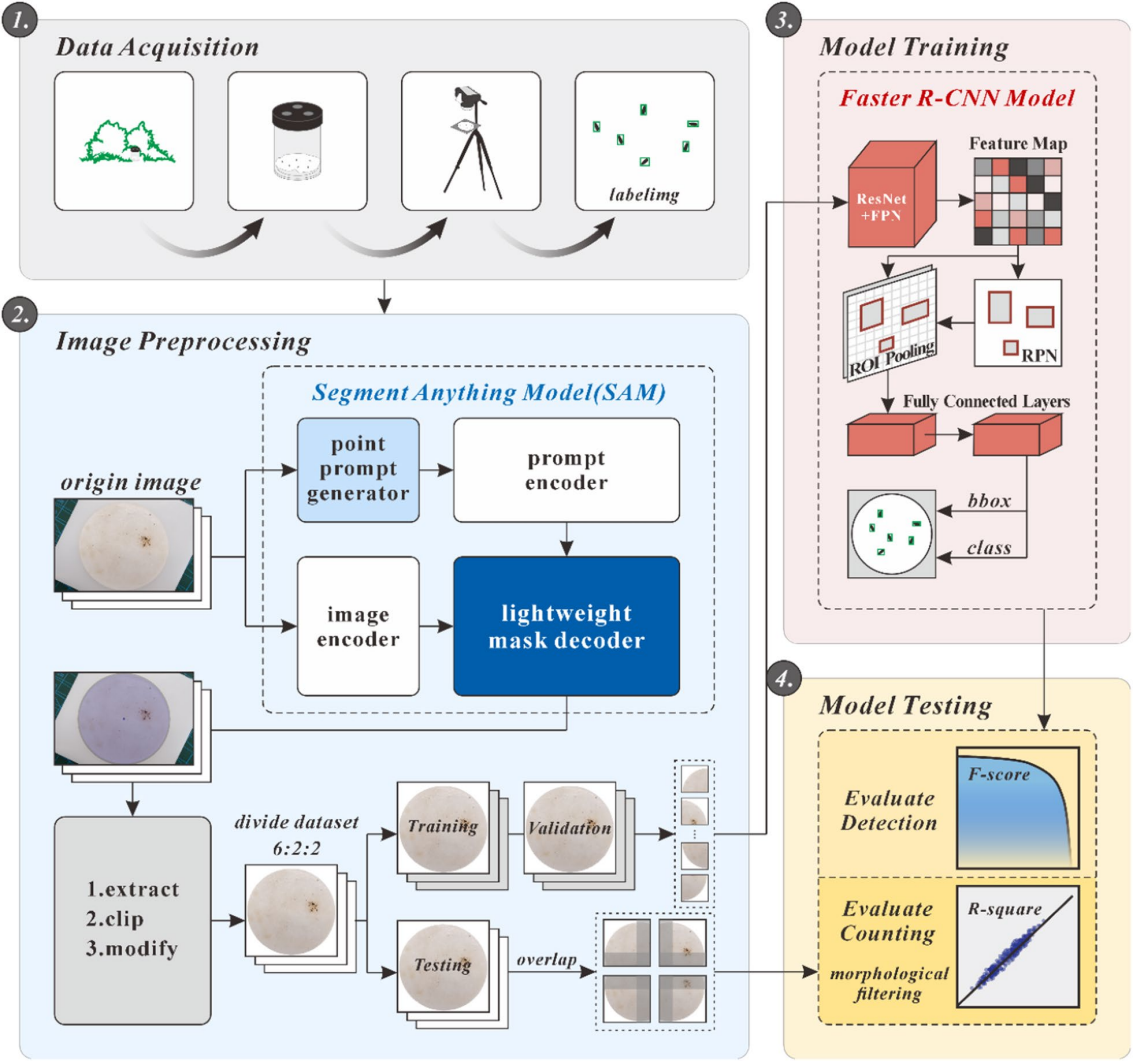
Experimental procedure

Figure 1 illustrates the study methodology. After images of *Ae. albopictus* mosquito eggs from field surveys were collected via a camera, manual labeling was carried out to create the necessary tags for training. The SAM was used to segment the images into the filter paper area and non-filter paper area, effectively removing the complex backgrounds and retaining only the filter paper area. We designed a point prompt generator to obtain a point prompt for SAM. Compared with other algorithms that require parameter training, the SAM can be used directly without training, demonstrating strong robustness. The dataset was subsequently divided into training, validation, and testing sets at a 6:2:2 ratio, and the training and validation sets were processed through segmentation. Then we trained a Faster R-CNN model based on image slices. Finally, the tile overlapping method was used to identify *Ae. albopictus* mosquito eggs, and morphological filtering was applied to count the identified eggs.

### *Aedes albopictus* mosquito egg collection

The ovitrap is a plastic container with a capacity of approximately 200 ml [26] and is equipped with a black lid with holes. This well-designed device not only offers a ventilated and shaded environment for mosquitoes but also prevents their escape once they enter. We added water to the container, which attracts mosquitoes to lay their eggs on filter paper [54]. The ovitrap is typically placed in bushes near human populations and sheltered from wind and rain to collect mosquito eggs. Such an environment, along with the presence of small water bodies in the ovitrap, is more suitable for *Aedes* mosquitoes to lay their eggs. The wild-strain *Ae. albopictus* mosquito eggs collected in this study were obtained from the Minhang District of Shanghai, China, with the collection period spanning from June to August 2023.

### Image acquisition

**Fig. 2:**
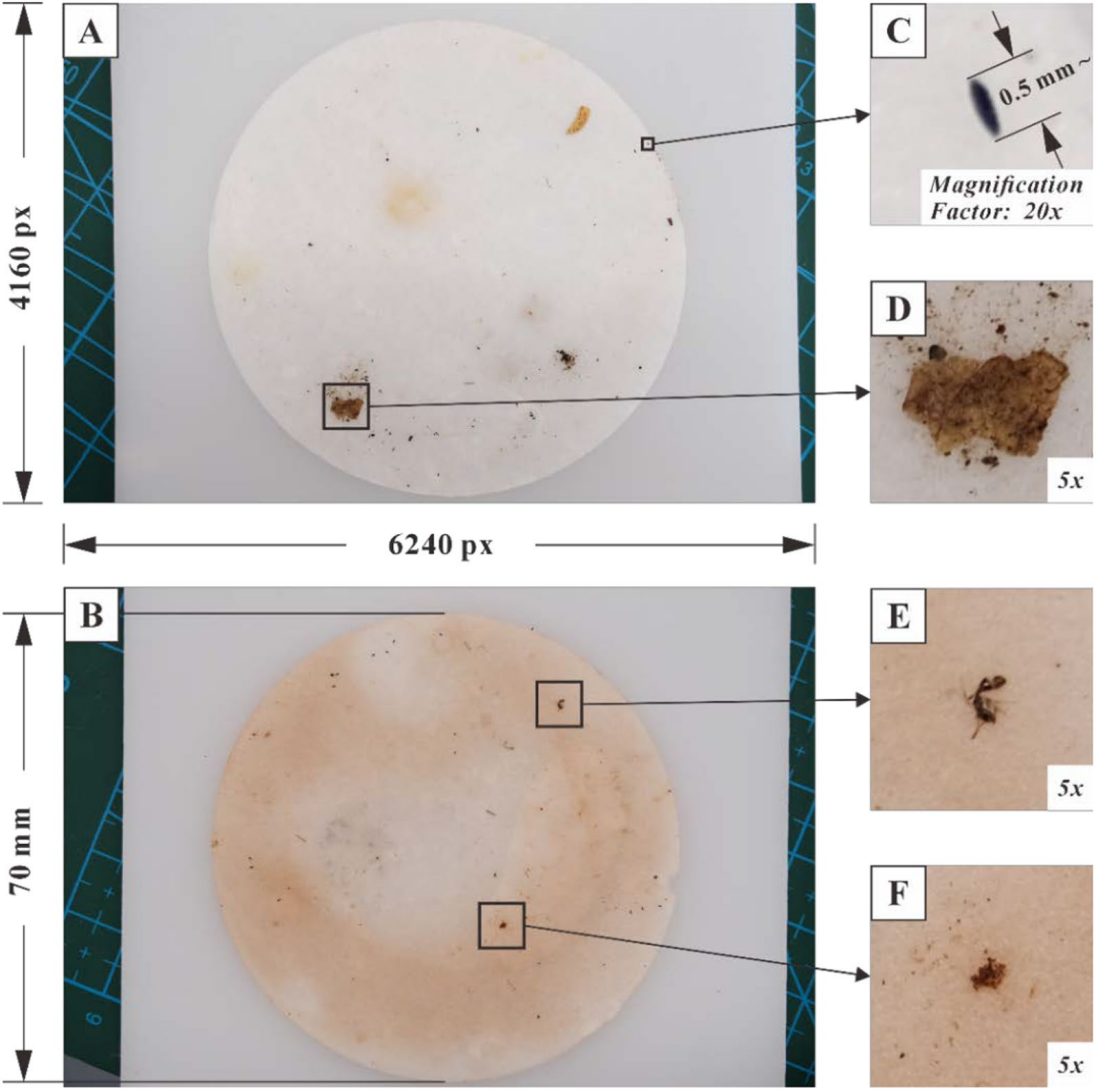
Overview of *Ae. albopictus* mosquito egg images. **A** Full view of the image. **B** Darkened filter paper with slight damage to the edges. **C** *Ae. albopictus* mosquito egg. **D** Impurities: plant debris. **E** Impurities: other insects. **F** Impurities: gravel

The images of *Ae. albopictus* mosquito egg filter papers were captured with the aid of a camera and tripod. A Fujifilm X-S10 mirrorless digital camera with 26.1 million pixels, equipped with an autofocus and image stabilization macro lens and a tripod, was used to form the image acquisition system. To prevent the filter paper from wrinkling, it was placed on a horizontal plastic board (or other flat surfaces) during shooting. This ensured that the filter paper was on the same focal plane, allowing each egg to be clearly visible in the images. A total of 1729 JPG format images were collected in this study, with each image measuring 6240 × 4160 pixels and an approximate file size of 10 megabytes. Compared with laboratory strains, the objects on the filter paper from wild strains are more complex, including *Ae. albopictus* mosquito eggs, and also contain a large amount of impurities such as plant debris, gravel, and other insects. The same applies to the background area beyond the filter paper. The color of the filter paper might also darken due to various factors, including the growth of bacteria or fungi, and prolonged exposure to sewage (Fig. 2).

### Dataset construction

We constructed a dataset of images of *Ae. albopictus* mosquito eggs from the wild strain. On the basis of the collected images of the *Ae. albopictus* mosquito eggs, we used LabelImg [55], a software widely applied in annotating images for object detection, to manually annotate the eggs in the images, creating egg labels in Pascal VOC format. The details of these annotations were stored in XML format, with the “name” field consistently labeled “egg.” We eliminated targets that were difficult to confirm manually to ensure that the objects being targeted were all correct. Additionally, we used BG-Trap for monitoring within the same research area, and the monitoring results included only *Culex pipiens pallens* and *Ae. albopictus*, which is consistent with the conclusions of relevant studies [56]. The eggs of these two mosquito species show distinct differences. The *Cx. pipiens pallens* mosquito eggs are conical in shape and are laid in rafts on the water surface. In contrast, *Ae. albopictus* mosquito eggs are generally elliptical and are laid individually at the bottom of the water. Furthermore, we also identified the adult mosquitoes collected in the ovitraps, and the results showed that they were all *Ae. albopictus*. These findings indicate that there are no other mosquito species in the research area whose eggs are similar to those of *Ae. albopictus.* Through Python code, the manually annotated dataset was divided into a training set (60%), a validation set (20%), and a testing set (20%).

### Image preprocessing

#### Extracting the region of interest

Given the diversity of image acquisition environments and the complexity of backgrounds beyond the filter paper, this study employs the SAM to extract the filter paper part of images. This approach addresses complex backgrounds and reduces their interference with the detection of mosquito eggs. The SAM is a versatile image segmentation model based on a vision transformer architecture, capable of segmenting a wide variety of objects without the need for task-specific training. Moreover, the SAM supports three types of prompts—point, box, and text—making it suitable for new tasks and capable of zero-shot learning, thereby enhancing its ability to handle complex backgrounds.

**Fig. 3:**
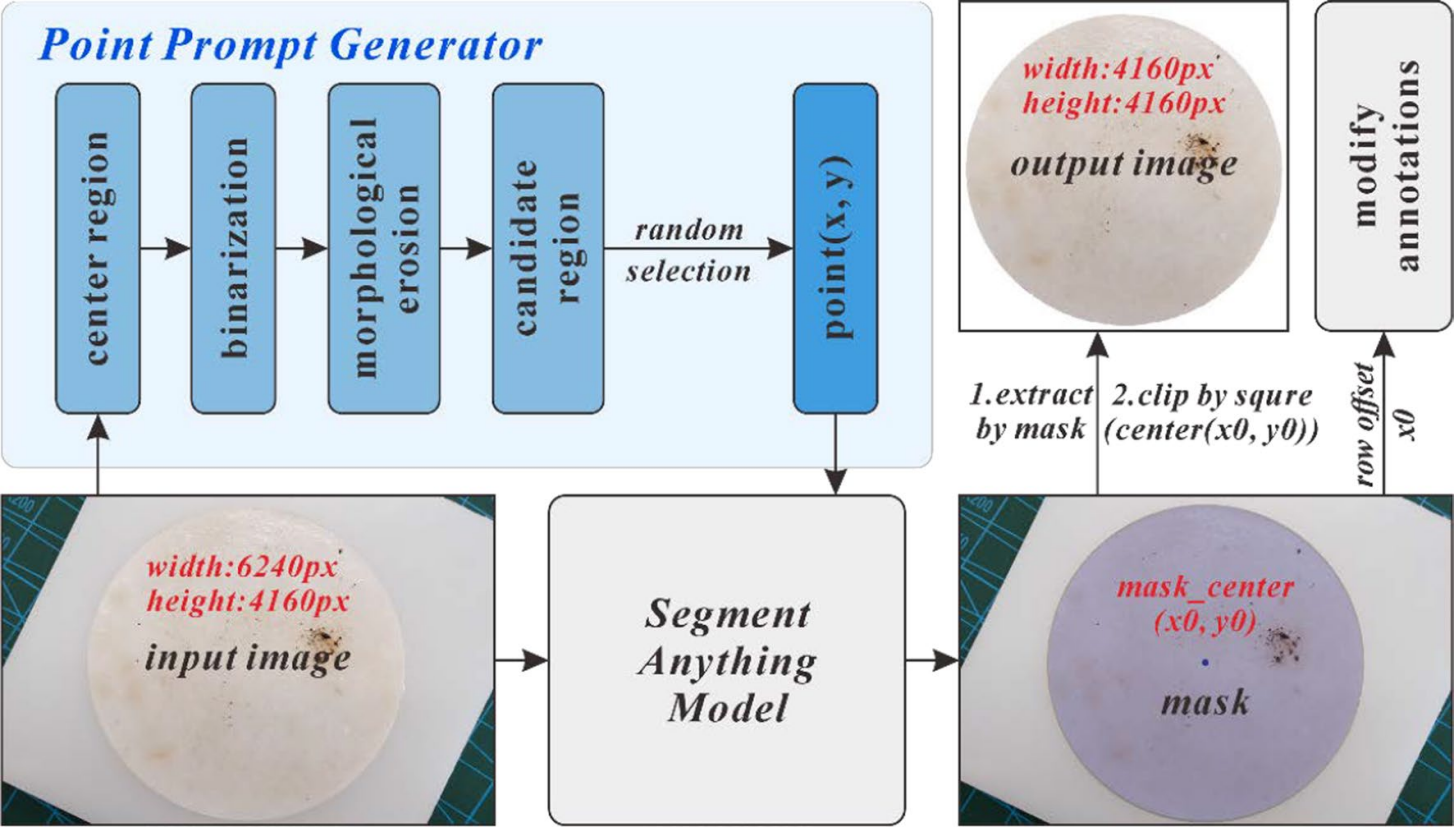
Process of extracting the region of interest

In this study, we developed a point prompt generator for obtaining a point-type prompt. The basic principle is as follows (Fig. 3): First, the width and height of the input image are extracted, and a square region with a side length of 500 pixels is determined in the center. The size of the square is adjustable, but should be small enough to be located within the filter paper. This square area is then binarized. To minimize the impact of the binarization threshold on the results, an erosion operation is performed to enlarge the pixels occupied by impurities, making the point candidate area more precise and improving robustness. Finally, one point is randomly selected from the candidate area to serve as the prompt. Using the point prompt in the SAM, a mask of the filter paper and the center coordinates of the mask (*x*_0_, *y*_0_) is obtained. The image is then cropped into a square centered on the filter paper with dimensions of 4160 × 4160 pixels, maintaining the same height as the original image. Subsequently, the original annotations are modified by subtracting the offset (*x*_0_ − 2080) from the horizontal coordinate *x*_0_, aligning the annotations with the cropped image.

#### Image tile

**Fig. 4:**
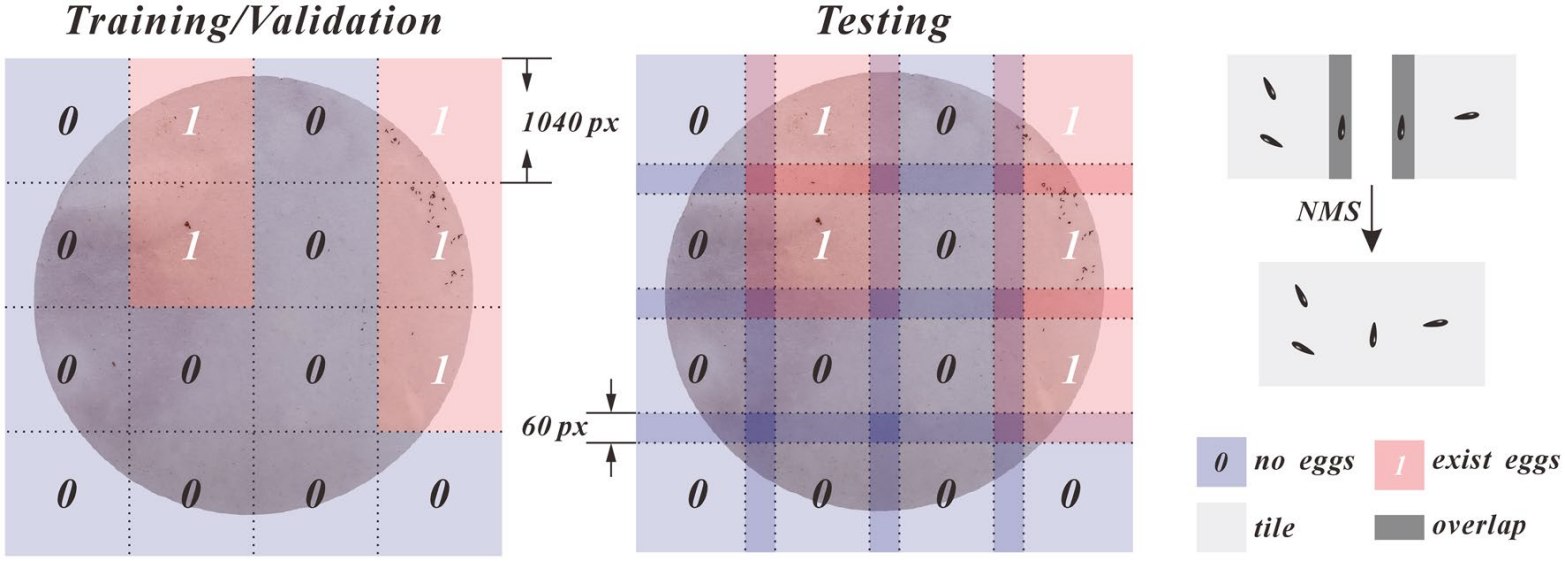
Image segmentation

In the Microsoft Common Objects in Context (MS-COCO) evaluation metrics, objects with dimensions smaller than 32 × 32 pixels are defined as small objects [57]. Owing to the limited classification information provided by small objects and the higher precision required for localization, improving detection accuracy is challenging. Generally, a smaller proportion of the detection target in the image results in lower detection accuracy [58]. To increase the pixel ratio of mosquito eggs, we divided the images of the training and validation sets into non-overlapping tiles (with a size of 1040 × 1040 pixels) in both the horizontal and vertical directions, and removed the tiles with “fragmented” or no eggs (Fig. 4). After division, the training and validation sets contain 8844 and 2922 images, respectively, maintaining the original 6:2 dataset split ratio.

The use of non-overlapping tiles can prevent the same eggs from appearing in multiple images, thus reducing data redundancy. However, the non-overlapping segmentation method may result in eggs at the edges being fragmented, which might lead to them being ignored or counted repeatedly during the identification process (we exclude these eggs during training). Therefore, when testing the model’s identification capabilities with a test set, we use a tile overlapping method (Fig. 4), which is used to divide the images into overlapping slices. Unlike direct segmentation, this method extends 30 pixels outward from the segmentation point to ensure that when an egg is partially segmented in a tile, it can be fully presented in other tiles. The size of expended pixels depends on the average length of eggs. During the testing phase, both the input and output are complete images, with the segmentation and extending processes performed automatically by the Python code. The code records the coordinates of the prediction boxes on each tile and then restores these prediction boxes back to their original positions in the complete image. This eliminates duplicate predictions with an IoU greater than 0.5 using non-maximum suppression (NMS) to avoid repeated identification of the same eggs.

### Model training and testing

#### Training

This study employs the advanced Faster R-CNN model, which is particularly effective in identifying small target objects [59], to identify the *Ae. albopictus* mosquito eggs. This model evolves from R-CNN and Fast R-CNN, utilizing the Region Proposal Network (RPN) to replace the selective search (SS) algorithm, achieving end-to-end training. The model consists of four modules: Convolutional Layers, Region Proposal Network, ROI [region of interest] Pooling, and Classification. Convolutional Layers are used for feature extraction, with common backbones including VGG16, ZFNet, and ResNet; the Region Proposal Network generates candidate region boxes; ROI Pooling resizes the feature matrix of candidate regions to a uniform size of 7 × 7, thus not limiting the size of the input image; Classification is used for object categorization. During the feature extraction process, lower feature layers have a higher resolution and contain rich positional and detail information but limited semantic information. Conversely, higher feature layers are rich in semantic information but may lose details, thus potentially missing small object detections. This research combines the feature pyramid network (FPN) [60] with ResNet50 [61], which is more effective for small objects, as the backbone of the model. The FPN enhances the model’s detection capabilities for small objects by upsampling and integrating features, thereby transferring the semantic information from higher feature layers to lower ones, thus improving the model’s ability to detect small objects.

We input the dataset into the Faster R-CNN ResNet50 FPN model and trained the model using the stochastic gradient descent (SGD) optimizer. The learning rate, momentum, weight decay, and batch size were set as 0.005, 0.9, 0.0005, and 8, respectively, for a total of 20 training epochs. The learning rate was decreased once every three epochs, with a decay rate (gamma) of 0.33. This learning rate decay mechanism helps enhance the stability of the training process, prevents the model from falling into local minima, and aids in optimizing the model more effectively. The training effectiveness of the model is assessed through the convergence of the loss value and learning rate, as well as the mean average precision (mAP) on the validation set.

#### Testing

After the model training, we use the test set to evaluate the model’s generalization ability. The tile overlapping method is employed for identifying the test set, and morphological filtering is used to remove impurities. Since mosquito eggs are generally similar in size [62], we filter the prediction boxes on the basis of the morphology of the mosquito eggs, eliminating those with areas less than 200 pixels or greater than 900 pixels and those with aspect ratios greater than 4, to further improve the model’s detection accuracy. The detection performance of the model is evaluated via precision, recall, and F1 score [63], with the values of these three indicators ranging from 0 to 1. A value closer to 1 indicates better quality of the model. The F1 score, which combines precision and recall, is often used to assess model quality. In this study, we calculate the precision, recall, and F1 score at different prediction probabilities, selecting the prediction probability corresponding to the maximum F1 score as the threshold for the output results during detection. In addition to assessing the model’s identification ability, we also counted the actual number of eggs and the predicted number of eggs in each image of the test set, and used *R*-squared and root mean square error (RMSE) to evaluate the model’s counting ability.

### Operating environment

In this study, dataset partitioning, image segmentation, the tile overlapping method, and morphological filtering were implemented via Python code. The hardware used for the experiments was an NVIDIA GeForce RTX 3080, and the software environments included Python 3.9.12, Torch 2.0.0, Torchvision 0.15.1, CUDA 11.7, and OpenCV 4.6.0.

## Results

### Model training

**Fig. 5:**
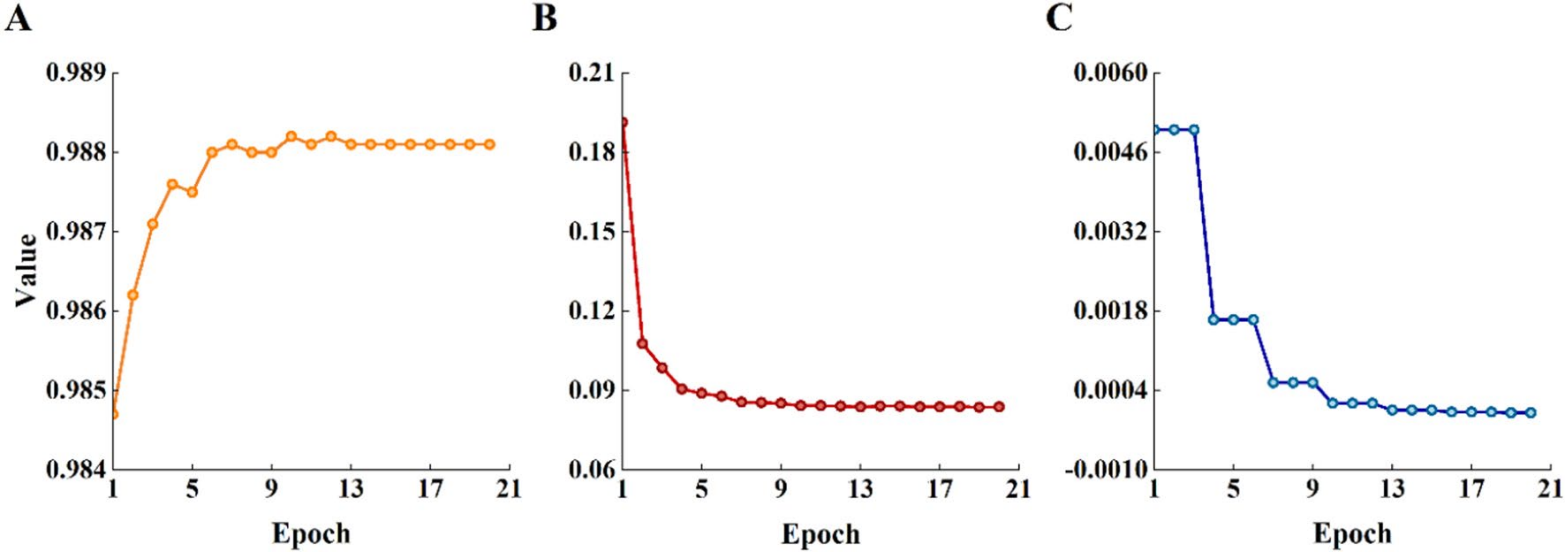
Model training process. **A** mAP. **B** Loss. **C** Learning rate

After 20 epochs of training, the model’s learning rate decreased from 0.005 to 0.000006 (Fig. 5A), with a decay every three training epochs. The loss value decreased from 0.1914 to 0.0839 (Fig. 5B), rapidly decreased in the first six epochs, and then gradually converged. The overall mAP increased with the number of training rounds, finally stabilizing at approximately 0.9881 (Fig. 5C). The gradual convergence and stabilization of the loss value and learning rate at lower levels indicate that the model’s predictions are increasingly consistent with the actual results, proving the reliability of the training outcomes. The mAP’s gradual stabilization at a high level demonstrates the strong recognition capabilities of the model developed in this study.

### Model testing

**Fig. 6:**
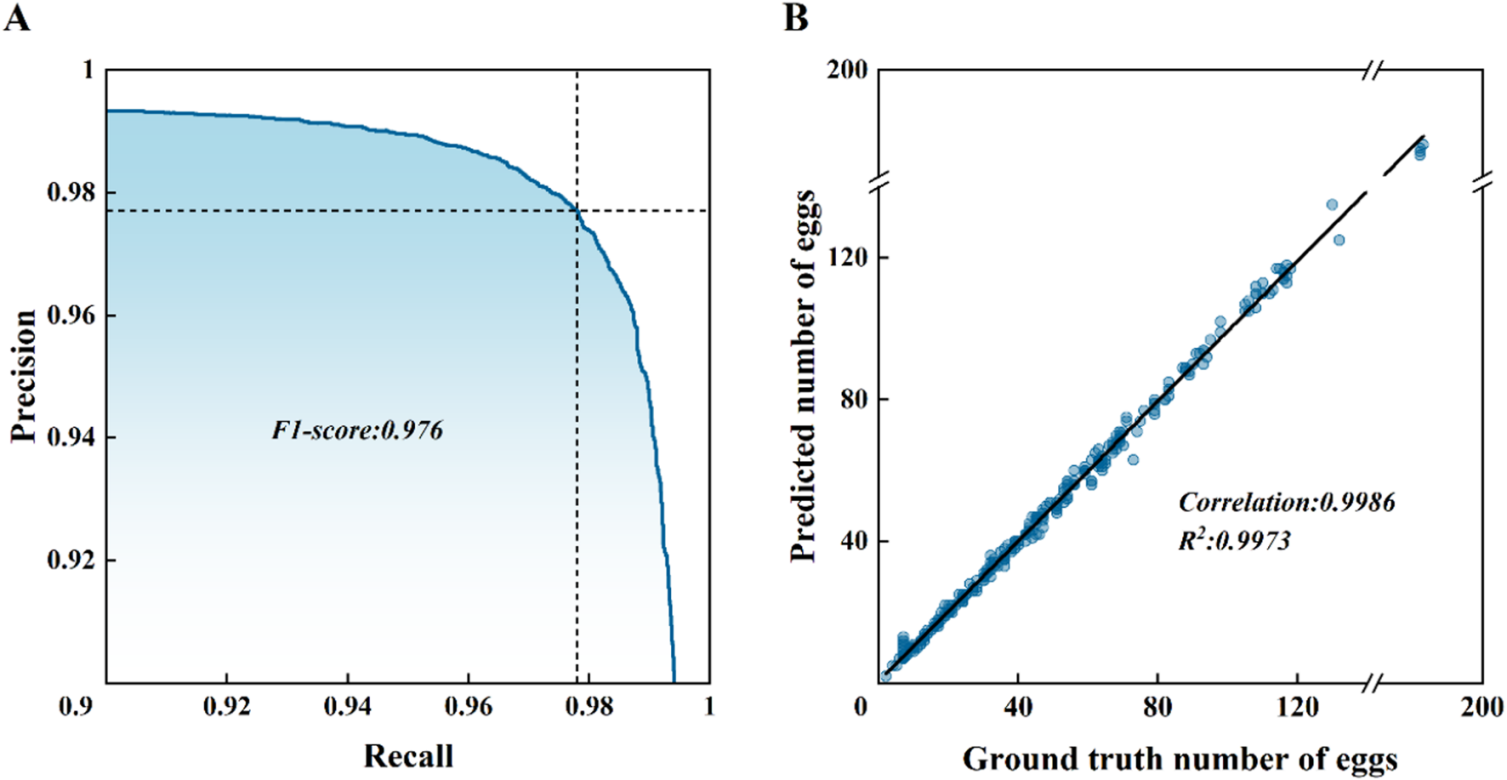
Evaluation of model identification and counting capability. **A** Precision–recall curve. **B** Regression curve

We segmented the images into 16 overlapping tiles, input them into the model for identification, and stitched the results after screening through mosquito egg morphology for the output. We calculated precision, recall, and F1 score, and plotted the precision–recall (P–R) curve for the evaluation of recognition ability (Fig. 6A). The model performed best when the prediction probability threshold was set as 0.9116, achieving precision of 0.977, recall of 0.978, and an F1 score of 0.977. In terms of counting ability, among the 334 images in the test set, the actual number of eggs was 15,952, while the model’s count was 15,974. Regression analysis on the actual versus predicted egg counts for each image yielded an *R*^2^ value of 0.997 (Fig. 6B), Pearson correlation coefficient of 0.994, and RMSE of 1.742. Among the 344 testing images, 140 had recognition results consistent with the actual counts, and 321 had errors within three eggs, resulting in an average counting error of 3.7%.

## Discussion

**Fig. 7:**
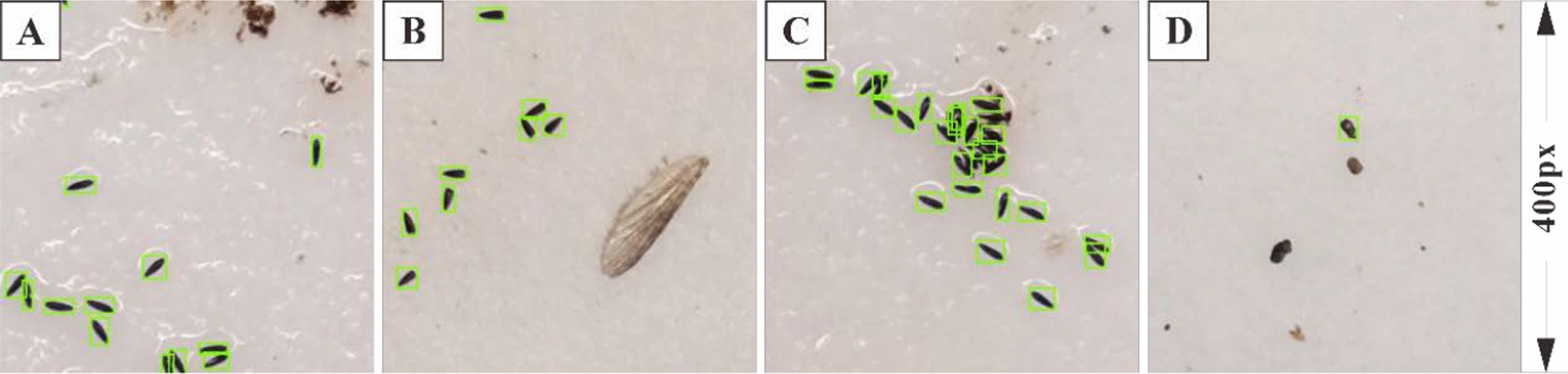
Egg identification cases. **A** Low- to medium-density *Ae. albopictus* mosquito eggs. **B** Incomplete mosquito eggs. **C** High-density *Ae. albopictus* mosquito eggs. **D** Impurities similar to eggs

Compared with similar studies [50, 52], our method shows significant improvement and demonstrates strong identification capabilities. During the identification process, the model can accurately recognize low- to medium-density *Ae. albopictus* mosquito eggs (Fig. 7A) and can even identify incomplete eggs (Fig. 7B). The model can also effectively eliminate large impurities (Fig. 7A). However, gravel that resembles the shape and color of mosquito eggs is difficult to remove and is likely to be misidentified as *Ae. albopictus* mosquito eggs (Fig. 7D), which negatively affects the precision of the model. Additionally, high-density egg clusters are prone to false negatives, leading to a decrease in the model’s recall (Fig. 7C). These two situations are the most challenging in both manual and machine-based identification and counting. Nevertheless, our method maintains high accuracy comparable to that of manual identification while significantly minimizing the processing time. Furthermore, our method exhibits strong counting capabilities, benefiting from its high precision and recall rates.

**Fig. 8:**
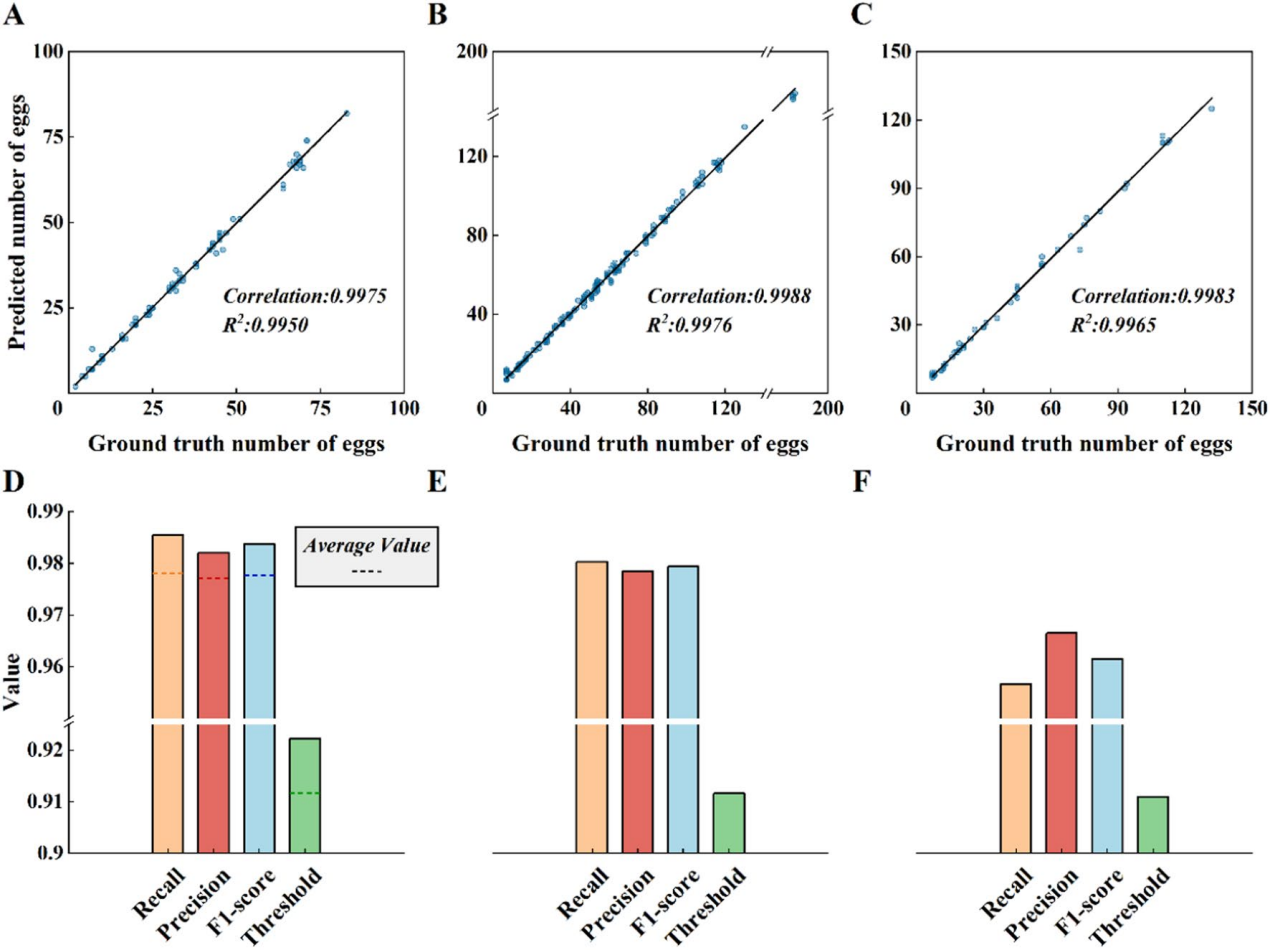
Evaluation of the applicability of the model in different scenarios. Regression curve: **A** easy, **B** medium, **C** hard. Model evaluation values: **D** easy, **E** medium, **F** hard

To thoroughly analyze the applicability of the model in different scenarios, we classified 344 test images into three categories based on their level of difficulty: easy (low to medium density of eggs with few impurities), medium (medium density of eggs with some impurities), and difficult (high density of eggs with a large number of impurities). Figure 8 shows the evaluation result. The images were divided based on the difficulty of manual labeling, with a total of 98 easy, 192 medium, and 54 difficult scenarios. In easy scenarios, the model demonstrated the best performance, which was close to that of the laboratory strains. These findings indicate that although our model is trained on wild strains, it provides sufficient information on *Ae. albopictus* mosquito eggs, showing strong recognition capabilities for eggs, which can be transferred for use in laboratory strains. Medium scenarios are the most common situation faced during practical applications. The model’s accuracy in medium scenarios is closest to that of the overall model without scenario differentiation. In difficult scenarios, precision, recall, F1 score, and detection thresholds all decrease. This is because, in such scenarios, a high density of eggs reduces the predictive probability of the eggs. Therefore, in practical applications, appropriately adjusting the thresholds according to specific scenarios can improve the model’s detection accuracy to a certain extent.

To verify the efficiency of the model in counting *Ae. albopictus* mosquito eggs, we compared its counting time using a magnifying glass with manual counting. We randomly selected three easy-, five medium-, and two hard-level images of *Ae. albopictus* mosquito eggs to closely match the dataset proportions. Table 1 presents the comparison results. The manual counting time is related not only to the number of mosquito eggs in the image but also to complex backgrounds and density levels, which can negatively affect the processing time of identification. When the effects of background and density are disregarded, the manual counting time is highly correlated with the number of eggs [29]. Complex background and denser eggs increase the counting time. However, the efficiency of counting is hardly affected when our method is applied, with an average detection time of 0.48 s per tile and 7.94 s for the entire image when the tile overlapping method is used. The coefficient of variation is less than 1%, demonstrating the model’s stability and consistency in performance. While ensuring high-quality counting results, our method is at least 10 times faster than the human–computer interaction method. In instances where the image contains a large quantity of densely distributed eggs against a complex background, the model demonstrates an efficiency improvement exceeding 50-fold.

**Table 1:** Temporal comparison of different methods

Class	Number of eggs (*n*)		Time (s)	
	Manual	Model	Manual	Model
Easy	69	69	102	8.09
–	83	83	138	7.88
–	42	42	83	7.89
Medium	22	22	84	7.98
–	79	77	156	7.94
–	182	177	388	7.92
–	39	40	124	7.97
–	17	17	79	7.82
Hard	132	125	453	8.03
–	56	57	183	7.88

We also designed experiments to verify the applicability of our method for broader use in practical scenarios. To explore the sensitivity of our method to camera specification, we examined the impact of lower specifications of image acquisition devices on the experimental results. We used OpenCV to downsample images, simulating images with different resolutions from various devices. The threshold for morphological filtering was adjusted proportionally with the resolution. Table 2 shows the results, which indicate that even when the image resolution is reduced to half of the original, the model’s F1 score remains stable. This indicates that our method is highly robust with low hardware requirements, making it suitable for most cameras, including mobile devices. Even at a quarter of the original resolution, the model’s accuracy and recall exceed 0.9. Moreover, when the image resolution is too low, transfer learning based on our model can significantly enhance identification accuracy. These experiments confirm the applicability of our method, demonstrating its potential for wide-ranging applications.

**Table 2:** Comparison of model performance at different image resolutions

Image resolution (pixels)	Downsampling ratio	Tile size	Overlapping size	Morphological filtering size (min, max, and ratio)	F1 score
4160 × 4160	1	1040	30	(200, 900, 4)	0.977
3120 × 3120	3/4	780	16	(112, 506, 4)	0.976
2080 × 2080	1/2	520	8	(50, 225, 4)	0.976
1040 × 1040	1/4	260	4	(12, 56, 4)	0.929

Despite the great contribution made by Arista-Jalife et al. [64], who increased the number of training samples from 916 to 18,320 by randomly rotating and moving images horizontally and vertically, the diversity of their training samples may remain insufficient, leading to a relatively weak generalization capability of the model. To address this problem, we collected 1729 images of wild-strain *Ae. albopictus* mosquito eggs in various complex scenarios to increase the diversity of the samples. However, the environment where *Ae. albopictus* mosquitoes live is complex; in further work, the training dataset can be further expanded to incorporate more scenarios. The recognition performance of the proposed model decreases in scenarios with high egg density and numerous similar impurities, which are also the most challenging situations for manual methods. To enhance the model’s performance in these scenarios, further work can be dedicated to increasing the number of image samples containing high-density mosquito eggs in various complex scenarios and negative sample labels of similar impurities to enhance the model’s recognition capability.

Garcia et al. [52] removed the black areas which had originated during the image acquisition with the magnification lens before training the model, which provides valuable insight for the image preprocessing workflow. We improved this operation by utilizing SAM, which concentrates on the region of interest, significantly reducing interference from complex backgrounds. However, a limitation of this method is that the prompts required by SAM need to be provided manually. To address this, we designed a point prompt generator, allowing for batch processing with a single manual parameter adjustment. Future research efforts can be dedicated to the automated point prompt parameter selection for different batches of images.

## Conclusions

To overcome the challenge of precise and efficient identification of mosquito eggs when monitoring the density of *Ae. albopictus* using ovitraps in wild environments, this study constructed an image dataset of *Ae. albopictus* mosquito eggs from wild strains and optimized the image preprocessing process using SAM. We trained a Faster R-CNN ResNet50 FPN model specifically for small target detection based on image segmentation, employed a tile overlapping method to identify *Ae. albopictus* mosquito eggs, and removed impurities through morphological filtering. The final model demonstrated strong performance in the identification and counting of *Ae. albopictus* mosquito eggs. The efficiency of this method is at least 10 times that of the human–computer interaction method, and even more than 50 times greater in complex situations, making it highly effective for identifying and counting eggs in wild strains with complex backgrounds.

## Data Availability

The data described in this article can be freely and openly accessed at https://figshare.com/s/06269306f9aa95ce8d63.
